# Supplementary material for: Complexome profiling of the Chlamydomonas psb28 mutant reveals TEF5 as an early PSII assembly factor
Source: Plant Cell. 2025 Mar 17;37(6):koaf055. doi: 10.1093/plcell/koaf055 (PMC12164590; doi:10.1093/plcell/koaf055)
Supplement: koaf055_Supplementary_Data [file koaf055_supplementary_data.zip › psb28_interactive.zip!/plots/intensity/qualityPlots/peptides/_Cre17.g722750.t1.2.html]

### Cre17.g722750.t1.2

Trivial name:   
SeqCoverage [median]: 33.30  
Mass: 38.98 kDa  
  
 

| Sequence | MissedCleavage | Mass [Da] | wtIntensity | muIntensity | Score |
| --- | --- | --- | --- | --- | --- |
| EAGVSLSR | 0 | 817.43 | 0.00 | 4390.85 | 106.36 |
| GASQESELDFVPR | 0 | 1433.68 | 0.00 | 1841.80 | 98.18 |
| HTLYVQHEVR | 0 | 1280.66 | 1363.42 | 3972.68 | 148.56 |
| KGVSVGAR | 1 | 772.46 | 600.69 | 0.00 | 72.49 |
| KVLYNDETDLLEYPADDGAR | 1 | 2296.09 | 0.00 | 1284.85 | 32.80 |
| LLGGGTLTASYDAAAR | 0 | 1535.79 | 0.00 | 1221.37 | 53.97 |
| QGVDAAELTSTGR | 0 | 1303.64 | 357.54 | 1256.91 | 116.55 |
| SSLIPSLK | 0 | 843.51 | 0.00 | 3794.62 | 97.80 |
| VIDGQADVGASLK | 0 | 1271.67 | 0.00 | 1267.04 | 70.98 |
|  |  |  | 2321.7 | 19030.1 |  |
